# Serological Survey and Molecular Typing Reveal New *Leptospira* Serogroup Pomona Strains among Pigs of Northern Italy

**DOI:** 10.3390/pathogens9050332

**Published:** 2020-04-29

**Authors:** Cristina Bertasio, Alice Papetti, Erika Scaltriti, Silvia Tagliabue, Mario D’Incau, Maria Beatrice Boniotti

**Affiliations:** 1 National Reference Centre for Animal Leptospirosis (NRCL), Istituto Zooprofilattico Sperimentale della Lombardia e dell’ Emilia Romagna “Bruno Ubertini”, via Bianchi 7/9, 25121 Brescia, Italy; 2 Risk Analysis and Genomic Epidemiology Unit, Istituto Zooprofilattico Sperimentale della Lombardia e dell’ Emilia Romagna “Bruno Ubertini”, Strada dei Mercati 13/a, 43126 Parma, Italy

**Keywords:** Leptospirosis, pig, MAT, real-time PCR, genotyping, epidemiology

## Abstract

Swine act as both maintenance and incidental hosts of pathogenic *Leptospira* spp. Here, a serological test was performed on 131,660 pig sera collected between 2002 and 2017 from 4715 farms in Northern Italy. A positivity rate of 13.05% was determined. Australis was the most frequently identified serogroup (77.29%), followed by Pomona (18.47%), Tarassovi (1.51%) and Icterohaemorrhagie (1.40%). Culture isolation and real-time Polymerase chain reaction (PCR) were carried out on 347 kidneys and 470 clinical samples, respectively. Overall, 133 strains were cultured successfully and 43 randomly chosen isolates were identified as serogroup Pomona. Multi-locus sequence typing (MLST) revealed that 41 isolates and 8 DNA extracted from biological samples belonged to sequence type 140. Using a multiple-locus, variable-number tandem repeat analysis, 43 samples produced identical profiles but, after 2014, three new *Leptospira interrogans* serogroup Pomona genotypes were observed. Interestingly, two isolates showed new MLST profiles and an unclassified identification by monoclonal antibodies. The 16S rRNA gene sequencing clustered them into *L. kirschneri* species and a core genome MLST analysis revealed an allelic identity of 96% compared with Mozdok strains. Genotyping allowed us to discriminate leptospires and to identify new emerging strains. The accurate identification of infective strains is required for formulating preventive methods and intervention strategies.

## 1. Introduction

Leptospirosis is the most widespread zoonosis worldwide and it is caused by an infection with any of the pathogenic members of the genus *Leptospira*. While, in theory, any pathogenic *Leptospira* may infect any animal species, leptospirosis is a disease that shows a natural nidality, and each serovar tends to be maintained in specific maintenance hosts. For example, serovar Bratislava is associated with pigs and horses, Canicola is commonly found in dogs, Icterohaemorrhagiae in rats, Pomona in pigs and Hardjo in cattle [1,2,3]. Leptospires persist in the kidneys of carrier animals and are excreted in urine and genital fluids [3,4,5,6]. In this way, they are spread through the environment where they can survive for long periods, contaminating the surface water, soil and muddy areas [2,3,4,5,7]. Chronically infected animals may remain carriers over years and act as reservoirs for the infection of other animals and humans [3,5]. Despite rat being the main reservoir for these bacteria, many other mammals can act as carriers. Swine, for example, act as a maintenance host for leptospires and are a possible source of human and domestic animal infections [3,5]. Historically, pigs act as a maintenance host for the serovars Bratislava, Pomona and Tarassovi, while among the incidental serovars, the most important in pigs are those belonging to the Icterohaemorrhagiae, Canicola and Grippotyphosa serogroups [3,4,5]. 

In Italy, swine act commonly as carriers for serovar Bratislava, belonging to the Australis serogroup, and serovar Pomona, belonging to the Pomona serogroup. Additionally, serovar Tarassovi causes commonly incidental infections [8]. Endemic infections in swine herds generally remain subclinical, and the only clinical symptoms are reproductive disorders, such as late-term abortions, and increases in mummified, stillborn and weak piglets. However, leptospires can also cause severe diseases depending mainly on the infecting serovar and the age of the animal [4]. Once the infective agent has entered a farm, it spreads very easily, mostly among fattening pigs, both through direct (contaminated urine) and indirect (infected feed, water and environment) contact [8]. Vaccination, therapy and farm management can all be used to limit the spread of infection in a breeding herd. 

Until 2010, in Italy, a trivalent vaccine against the serogroups Australis, Pomona and Tarassovi was available for swine. Nevertheless, very few breeders have adopted vaccination practices, primarily because the risk of leptospirosis is poorly understood by farmers and because the treatments and/or control strategies for other more virulent diseases are more important to the farm’s economy. In 2011, vaccinations were completely abandoned because no commercial vaccine was available for pigs. Starting from that moment, the main infection control strategy was the management of the breeding herd through the prevention of direct or indirect contact with free-living vectors or other domestic stock. Strong monitoring and surveillance systems are needed to better understand the disease epidemiology, and strict biosecurity tools should be applied to limit the transmission of the infective agent. Leptospirosis is not included in the Office International des Epizooties (OIE) list of notifiable terrestrial and aquatic animal diseases; however, in Italy, it is considered, by the current rules, a notifiable infection [9]. In cases of clinical suspicion, which are confirmed by a serological examination, the outbreak must be officially reported to the authorities. The farm is seized and sanitary measures, aimed at the eradication of the infective agent, are implemented. The interruption of transmission through the isolation of the infected animals is the first action that must be implemented. Only when all the animals become seronegative may the infection be considered eradicated, and the restrictive measures can be removed [9,10]. This can take months, or even years, resulting in significant economic losses.

In the laboratory, serological testing is the most widely used means for diagnosing leptospirosis, and the Microscopic Agglutination Test (MAT) is the serological gold standard method reported by the OIE [11]. Ideally, antigens selected for the MAT should include representative strains of all serogroups known to circulate in the study region as well as those known to be maintained elsewhere by the host species being analyzed. 

The OIE indicates that strain isolation should be attempted from biological samples. Cultured strains can be studied in depth, and the serogroup and serovar status should be determined using polyclonal and Monoclonal Antibodies (mAbs), respectively [11]. Nevertheless, isolation does not contribute directly to acute diagnosis of leptospirosis because *Leptospira* grows slowly over weeks [12]. With the advent of molecular methods, the diagnosis of a *Leptospira* infection has become simpler and faster. Over the years, a number of real-time PCR-based methods have been described for the direct identification of *Leptospira* nucleic acids in biological samples, both for detection of pathogenic and environmental species [13,14,15,16,17,18,19,20]. Real-time PCR provides advantages over the classical conventional reference methods used to diagnose leptospirosis (such as MAT and isolation) including reduced turnaround and hands-on times, low carryover contamination risks and higher sensitivity and specificity levels.

Furthermore, in the last few years, genotypic classification has increasingly integrated traditional serological classification in a wide variety of bacterial species. For *Leptospira*, the classical classification into serogroups and serovars has been joined by molecular typing. Several molecular techniques, such as Multiple-Locus Variable-Number Tandem-Repeat (VNTR) Analysis (MLVA) and Multi-Locus Sequence Typing (MLST), aimed at identifying bacteria by examining individual genomic profiles, have been used to investigate the epidemiology of *Leptospira*. The MLVA method is a useful typing tool for identifying *Leptospira* genotypes by providing information on genetic relationships among isolates for the surveillance of *Leptospira* populations. Salaün and colleagues [21] developed an MLVA method that permits the discrimination of the three most common pathogenic species of *Leptospira* (*L. interrogans*, *L. kirschneri* and *L. borgpetersenii*) through the analysis of five loci of repetitive unit sequences (VNTR 4, 7, 10, Lb4 and Lb5).

In 2013, Boonsilp and colleagues [22] proposed an MLST scheme based on the sequencing of seven housekeeping genes. This protocol is optimized to work on isolate samples, but it is not effective on biological samples, owing to the poor bacterial load. Thanks to a new protocol published in 2016 by Weiss [23], it is now possible to perform MLST directly on DNA extracted from biological samples, overcoming the long and difficult isolation step. With Whole-Genome Sequencing (WGS), new genome-based analyses have been developed for bacterial typing. Very recently Guglielmini et al. [24] published a core genome MLST (cgMLST) scheme based on 545 highly conserved genes in *Leptospira*, increasing the discriminatory power necessary to distinguish isolates.

This study aims to provide a serological survey of the prevalence of *Leptospira* in pigs in Northern Italy and to characterize the circulating strains using innovative molecular techniques. Our goal is to understand the epidemiology of swine leptospirosis in this specific area and to identify possible new emerging strains to address preventive and control measures and to reduce the risk of infection in swine herds.

## 2. Results

### 2.1. Microscopic Agglutination Test (MAT)

Using MAT, 17,184 out of 131,660 sera collected from 4715 farms were identified as positive, with a seropositivity of 13.05% (cut-off ≥ 1:100). The minimum percentage of positive sera was observed in 2006 (8.46%), the maximum one in 2012 (18.31%). Among the positive samples, 13,809 (10.49% of the total samples and 80.36% of the positive samples) tested positive for one serogroup, while 3375 (2.56% of the total samples and 19.64% of the positive samples) were positive for more than one serogroup. Antibody titers against the serogroup Australis occurred in 8.11% of the total samples and 77.29% of the single positive samples, followed by Pomona (1.94% and 18.47%, respectively), Tarassovi (0.16% and 1.51%, respectively), Icterohaemorrhagiae (0.15% and 1.40%, respectively), Sejroe (0.07% and 0.67%, respectively), Ballum (0.03% and 0.32%, respectively), Canicola (0.02% and 0.22%, respectively) and Grippotyphosa (0.01% and 0.12%, respectively) (Table 1). Using the cut-off ≥ 1:200, the Australis-positive samples decreased to 66.75%, the Pomona-positive samples increased to 29.28%, and the other serogroups maintained similar values (data not shown).

For positivity to more than one serogroup (Table 2), the predominant combination was represented by Australis-Pomona, having a prevalence of 54.72% (1847 out of 3375 multiple-positive sera) followed by Australis-Icterohaemorrhagiae-Pomona with 16.91% (571/3375), Australis-Tarassovi and Australis-Icterohaemorrhagiae (6.40% and 6.34%, respectively). The Icterohaemorrhagiae-Pomona combination was found in 2.75% of samples, while other combinations were revealed at levels < 2%. Australis was present in 3156 (93.51%), while Pomona was present in 2855 (84.59%), of 3375 multiple-positive sera.

**Figure 1:**
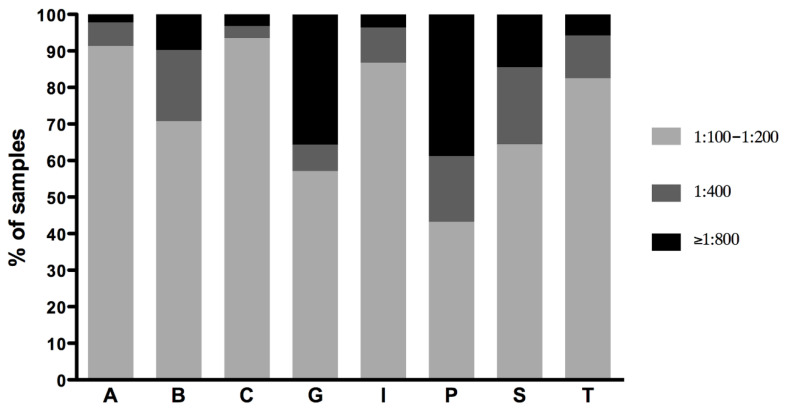
Distribution of MAT titers of samples reacting to one serogroup. Titers were grouped as low (1:100–1:200), medium (1:400) and high (≥1:800). A, Australis; B, Ballum; C, Canicola; G, Grippotyphosa; I, Icterohaemorrhagiae; P, Pomona; S, Sejroe; T, Tarassovi.

The MAT titers of the single positive samples were generally low (1:100 or 1:200) for all the serogroups detected, except for the serogroup Pomona, which showed medium (1:400) or high titers (greater than 1:800) in more than 50% of the samples (Figure 1). In particular, more than 90% of the samples positive for the serogroup Australis showed low antibody titers, while those positive for Pomona showed mainly medium (17.99%) and high (38.79%) titers. Grippotyphosa-positive samples also showed high titers (≥1:800) in more than 35% of the samples (Figure 1). The detailed titer distributions of positive sera reacting to one serogroup are reported in Table 3.

Out of 4715 tested farms, a mean of 53.62% resulted as positive for the presence of pathogenic *Leptospira* (Table 4). A farm was considered positive if at least one pig was positive by MAT to one or more *Leptospira* serogroups. The percentage of farms with positive test results ranged between 22.84% (n = 140) in 2006 and 77.52% (n = 231) in 2012.

**Figure 2:**
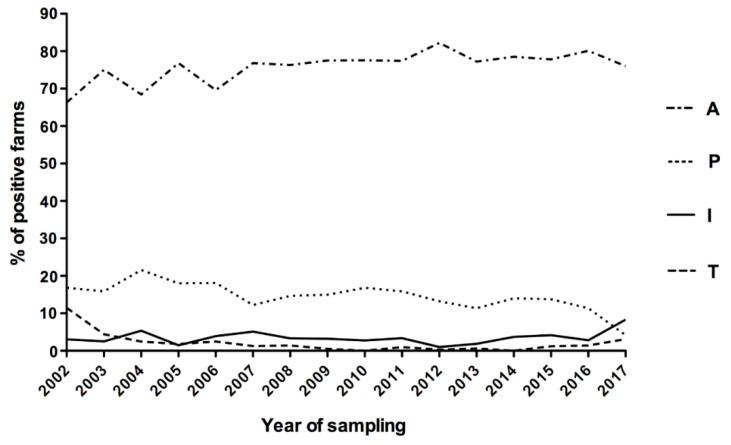
Trend in the main observed outbreaks between 2002 and 2017 (only sera positive for a single serogroup were considered). A, Australis; B, Ballum; C, Canicola; G, Grippotyphosa; I, Icterohaemorrhagiae; P, Pomona; S, Sejroe; T, Tarassovi.

Outbreaks caused by Australis were stable over the years, with a maximum value of 82.23% in 2012 and a minimum of 66.34% in 2002. This serogroup is responsible for a mean of 75.87% ± 0.15% (Confidence Interval 95%) of the outbreaks during the study period (Figure 2).

The second serogroup in terms of frequency of outbreak was Pomona, with a mean of 14.56% ± 0.31% (CI 95%) over the 16 years of the study with the maximum value observed in 2004 (21.58%) and the minimum one in 2017 (4.17%). Serogroup Icterohaemorrhagiae was responsible for a mean frequency of 3.52% ± 0.30% (CI 95%), with a peak of 8.33% in 2017. In 2017, an inversion occurred between the Pomona and Icterohaemorrhagiae serogroups: the first decreased to the minimum value (4.17%), while the latter increased to a new maximum level (8.33%). The mean frequency of Tarassovi was 2.10% ± 0.48% (CI 95%) but interestingly, its trend started from 11.49% in 2002 and became null in 2014. For Sejroe, a very low frequency was registered over the years, with values between 0% in 2014 and 2.84% in 2016 (mean of 1.64% ± 0.19%, CI 95%). For the other serogroups, the mean prevalence was less than 2% with rare exceptions during the considered time (Supplementary Table S1).

### 2.2. Molecular Detection, Isolation and Identification

Using real-time PCR, we detected pathogenic *Leptospira*l DNA in 102 out of 470 biological samples (21.70%). In total, 133 out of 347 samples (38.33%) were cultured successfully. The MAT carried out using the isolated strains as antigens against reference anti-sera showed that they were antigenically related to the Pomona serogroup. In total, 12 randomly chosen isolates (ID321, 331, 335, 340, 349, 354, 362, 385, 391, 430, 393 and 411) were subjected to serovar identification with mAbs. The reference strains Pomona and Mozdok 5621 reacted to mAb panels as expected (Table 5). Our isolates 321, 331, 335, 340, 349, 354, 362, 385, 391 and 430 reacted only to mAbs F43 C9 and F48 C6, suggesting that they belonged to the serovar Pomona of the *L. interrogans* species. The reaction profiles of isolates 393 and 411 were very similar to each other, but different from any other strain of the serogroup Pomona. If we consider an acceptable level of variability for the two dilutions used for the reaction titers, then all the mAbs, except for F48 C6 against which both isolates did not show any reactivity, indicated that they were related to the reference strain Mozdok 5621. The Tsaratsovo reference strain B 81/7 had reaction patterns for mAbs F48 C6 and F61 C7 that were similar to those of our isolates. However, the Tsaratsovo reaction patterns against mAbs F43 C9, F46 C9 and F58 C1 are unknown; therefore, they could not be compared with those of our isolates.

### 2.3. Genotyping Analyses

#### 2.3.1. MLST and MLVA Analyses

The genotypes of 43 randomly chosen isolates (including the 12 isolates tested for serovar determination) and the 8 biological samples (Supplementary Table S2) were determined. Using the MLST technique, 41 out of 43 isolates and all 8 DNAs extracted from biological samples were identified as ST140 and clustered with reference strain *L. interrogans*, Pomona st. Pomona (international reference strain) and st. Mezzano I (national reference strain). In total, 2 (ID 393 and 411) out of 43 isolates showed two new sequence types (STs), similar to ST117, typical of *L. kirschneri* Mozdok and also previously determined for reference strain 5621 present in the Istituto Zooprofilattico Sperimentale della Lombardia e dell’Emilia Romagna (IZSLER) collection. Compared with ST117 (pattern 13-25-15-22-33-18-23 for the loci glmU, pntA, sucA, tpiA, pfkB, mreA and caiB, respectively), the glmU gene of sample 393 showed the substitution 120 T > C, and the pntA gene had the substitution 410 A > G. The isolate 411, compared with ST117, showed the mutated pntA gene already found in isolate 393, while the glmU gene was the same as that of ST117 (allele 13). Both the sequences were submitted to the curators of the *Leptospira* database and alleles 75 and 85 were assigned to glmU and pntA, respectively. These new alleles defined two new STs, ST288 and ST289, for isolates 393 and 411, respectively.

**Figure 3:**
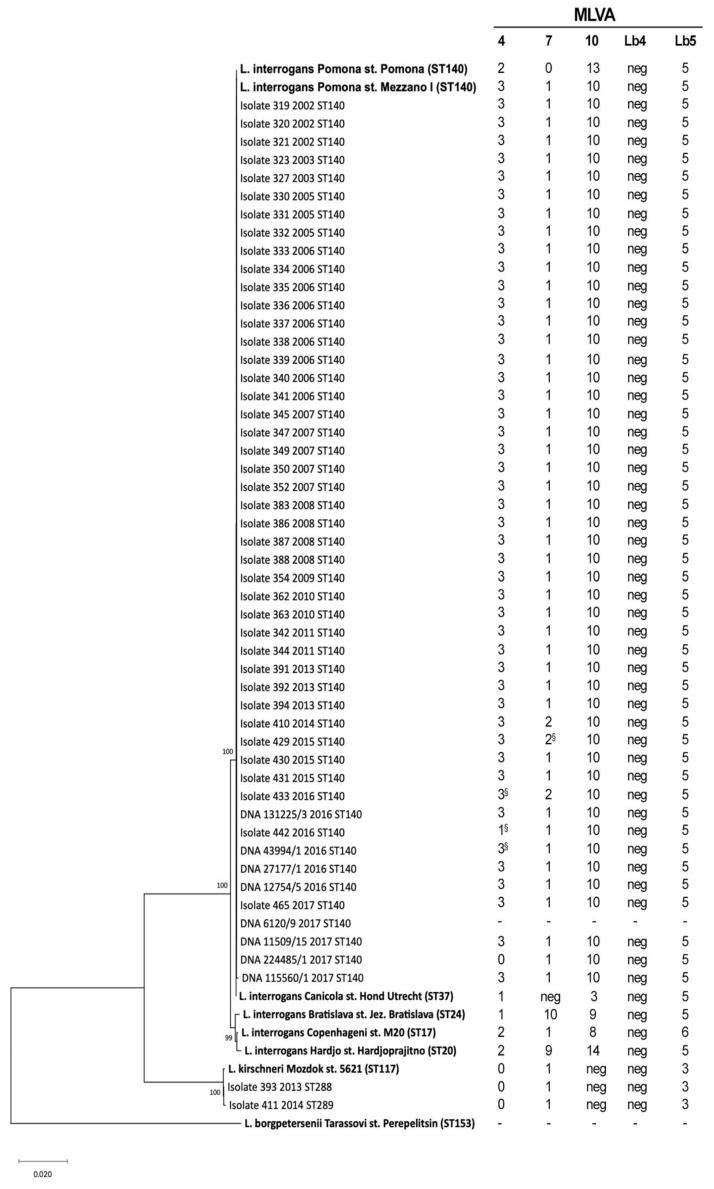
Phylogenetic tree based on concatenated sequences of the seven gene of the multi-locus sequence typing scheme (3111 bp) [22]. Samples are indicated with their unique IDs, the isolation year and their sequence types (STs). The names of reference strains (in bold) include the *Leptospira* species serovar and strain. The percentages of replicate trees in which the associated taxa clustered together in the bootstrap test (1000 replicates) are shown next to the branches. In the multiple-Loci Variable Tandem Repeat Analysis (MLVA) profile (on the right), the symbol “§” indicates the sequenced alleles.

As shown in Figure 3, 41 out of 43 isolates and all the DNA samples clustered together with the *L. interrogans* Pomona reference strains (Mezzano I and Pomona) having ST140, while the samples 393 and 411 clustered with *L. kirschneri* species near the reference strain 5621.

Moreover, the MLVA analysis revealed that 37 out of 41 isolates, belonging to ST140, and 6 out of 8 DNA samples, had the MLVA profile 3-1-10-neg-5 for the VNTR loci 4, 7, 10, Lb4 and Lb5, respectively. This profile was identical to that of the Italian reference strain (Mezzano I), but different from the international reference strain (Pomona) profile (2-0-13-neg-5) at the loci 4, 7 and 10 (Figure 3). Three isolates belonging to ST140 (one each from 2014, 2015 and 2016) showed a new genotype (3-2-10-neg-5), which differed from strain Mezzano I and from the other samples at the VNTR7 locus. One ST140 isolate from 2017 (ID 442) harbored the new genotype 1-1-10-neg-5. One extract from 2017 (224485/1) and genotyped as ST140 showed a new VNTR profile that was 0-1-10-neg-5. The new alleles were confirmed by sequencing (Figure 3). No results were obtained for sample 6120/9, although many amplification attempts have been made for each of the five VNTR loci (Figure 3). The isolates 393 and 411 showed the same genotype found in strain Mozdok 5621, which is 0-1-neg-neg-3 for the loci 4, 7, 10, Lb4 and Lb5 respectively.

Sequencing the 16S rRNA gene confirmed that strains 393 and 411 belonged to *L. kirschneri* species, in agreement with the previous results.

#### 2.3.2. Whole Genome Sequencing (WGS) and Core Genome MLST (cgMLST) Analysis

To clarify the genetic features of strains 393 and 411 belonging to the new STs, we performed WGS and a cgMLST analysis. Sequences of strain 393, 411, IZSLER 349/2007 and Mozdok 5621 were submitted to EBI database under the project number PRJEB36553 and are available under genome accession numbers from ERR4056312 to ERR4056315. The cgMLST analysis was performed on contigs of the strains 393, 411, IZSLER 349/2007 and Mozdok 5621 using the Bacterial Isolate Genome Sequence Database (BIGSdb) [25] (Table 6). The strains 393, 411 and IZSLER 349/2007 showed similarities with more than one cgST profile.

The closest cgSTs were queried against the BIGSdb to retrieve deposited isolates with the same genotypes. The results are reported in Table 7. 

The identities of strains Mozdok 5621 and IZSLER 349/2007, included in the cgST analysis as controls, were confirmed (Table 6 and Table 7). The isolates 393 and 411 showed the greatest similarity (96%) with deposited strains classified as *L. kirschneri* Pomona Mozdok (Table 6 and Table 7). We performed a cgMLST comparison (BIGSdb Genome Comparator [25]) among genomes of sequenced isolates (strain 5621, 393 and 411) and cgST-related strains present in the database (134|Vehlefans_2, 140|Vehlefans_3, 251|61H and 252|M36/05) (Supplementary File S1).

**Figure 4:**
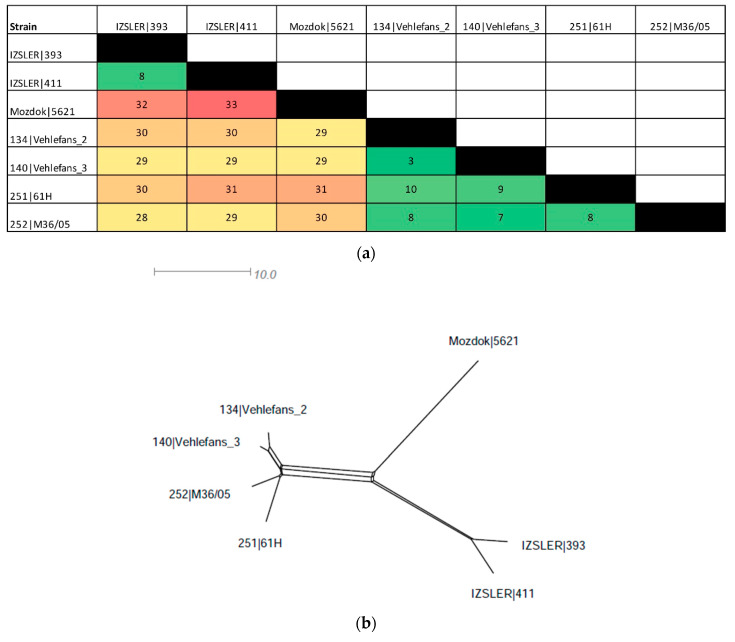
Distance matrix of the cgMLST of IZSLER isolates and cgST-related strains in the BIGSdb (**a**) and SplitsTree networks constructed using the distance values (**b**). (**a**) The color level of the distance matrix is proportional to the similarity value between strains, from green (high similarity) to red (low similarity). Incomplete loci were ignored in pairwise comparisons unless the locus was missing in an isolate. (**b**) Distance matrix visualized as SplitsTree networks.

In total, 17 and 19 out of 545 alleles were revealed as “new” in isolates 393 and 411, respectively. In total, 14 occurred in both isolates 393 and 411 (Supplementary Table S3). The distance matrix (Figure 4a) was used to build a distance tree using SplitsTree software ver. 4.15.1 [26] (Figure 4b).

The strains 393 and 411 were very similar, having only eight allelic differences. Compared with Mozdok 5621, they had 32 and 33 allelic mismatches, respectively (Supplementary File S1 and Figure 4) and showed from 28 to 31 differences when compared with the other serovar Mozdok strains retrieved from the database (134|Vehlefans_2, 140|Vehlefans_3, 251|61H and 252|M36/05) (Supplementary File S1 and Figure 4a). The SplitsTree network revealed three different strain clusters: the first contained the Mozdok strains retrieved from the BIGSdb (3–10 differences between each other), the second contained the IZSLER strains 393 and 411 (eight differences between each other) and the last contained Mozdok 5621, which was located on a separated branch (Figure 4b).

## 3. Discussion

The main objective of this study was to evaluate the epidemiology of infective *Leptospira* serovars in fattening pigs from farms in Northern Italy (Lombardy and Emilia Romagna regions). This territory has a very large pig population, with a mean of 5,300,000 animals, and contained 12,900 farms from 2007 to 2017 (data for 2002–2006 are not available), which produce pork for the entire country. In this area, the control of swine pathogens responsible for zoonoses, like *Leptospira*, is crucial to limit human and domestic animal infections, as well as to reduce the related economic losses linked to such diseases on the farms. Furthermore, the identification of the strains responsible for leptospirosis in pigs and their specific genotypes are essential for epidemiological analyses needed to adopt proper preventive measures and to better understand the etiology of the disease.

In this study, MAT, considered the gold standard and the most commonly used test, was used for serological identification. We tested 131,660 pig sera collected for 16 years. In 2002, there were 18,096 collected samples but the number progressively decreased each year to 2505 samples in 2017, except for a peak of 9330 samples in 2012. This trend may result from the strictness of the Italian national rules [9,10], in which the infection associated with a disease outbreak is only considered eradicated when all the affected animals become seronegative. This could require months or even years, and during this period, the herd is subjected to restrictive measures that are very penalizing and economically disadvantageous to the farm. Moreover, a better sampling strategy may have contributed to the reduced numbers of samples over the last few years. In fact, in the past, sampling had been carried out in all the livestock sectors, but in the last few years, perhaps for economic reasons, the trend has been to perform targeted sampling, mainly among reproductive or symptomatic animals. 

In the MAT analysis, to discriminate positive and negative sera, we considered a cut-off titer of 1:100, following the guidelines indicated by the OIE [11] and commonly used for international trade. More than 80% of the positive sera (relative to 10.49% of the total samples) tested positive for one serogroup, presumably indicating the real infecting serovar and the chronic stage of infection [7]. Instead, 19.64% of the positive sera reacted simultaneously with two or more serovars, indicating both cross-reactions of serovars of the same serogroup and the acute phase of infection [4,7]. In fact, paradoxical immune response, in which the predominant serogroup in MAT is unrelated to the infecting one, occurs in the acute phase of infection meaning that 2.56% (3375 out of 133,660 sera) of all pigs examined in this study were very likely in an acute stage of infection [7]. 

Considering both single positive sera and multiple positive sera, our results indicated a seropositivity of 13.05%, in agreement with other previous studies [27,28,29]. In particular, Bertelloni found a seroprevalence of 16.6% among slaughtered swine in North–Central Italy [27], Cerri reported a prevalence of 8.85% in swine sera collected in Italy from 1995 to 2001 [28], and Chiari and colleagues found an overall prevalence of 15.28% among wild boars in Northern Italy in a five-year period (2008–2013) [29]. In Brazil, the prevalence of seropositive pigs is 16.1% [30] and in Japanese herds it is 14.4% [31]. However, a very low prevalence (1.02%) has been found in the pig population of Poland [32].

Our data revealed that a mean of 53.6% of pig farms sampled in Northern Italy tested positive for leptospirosis, in agreement with the data reported by Bertelloni and colleagues [27], who observed a leptospirosis prevalence of 52.5% in farms located in North–Central Italy from September to December 2015. Interestingly, positivity among farms showed a wide variability, from a minimum of 22.8% in 2006 to a maximum of 77.5% in 2012, in accordance with the tendency over the last few years to perform targeted sampling on suspected cases. 

The most common serovars associated with swine infections in Italy are Pomona (serogroup Pomona), Tarassovi (serogroup Tarassovi), Bratislava (serogroup Australis) and Muenchen (serogroup Australis) [8]. Our results indicated that Australis is still endemic in pigs in Northern Italy, where swine acts as reservoir host for this serogroup, as in many countries and regions worldwide [4]. More than 90% of Australis-infected animals had low antibody titers (≤1:200); this is in agreement with previous studies that described serogroup Australis, specifically serovar Bratislava, as pathogenic for swine, although it usually causes a weak immune response and most of the chronically infected pigs have titers less than 1:100 [33,34,35]. Infections caused by Bratislava are characterized by mild clinical signs and are frequently associated with subfertility and litter-size reduction [36,37]. Unfortunately, since any Bratislava strains were successfully isolated, there is no data that allow us to define the specific infective serovar present on Australis-positive farms. It is probable that these infections were largely caused by *Leptospira* Bratislava, which is widespread among hedgehogs (*Erinaceus europaeus*) [38] and was found in wild boars in Parma Apennines in Italy [39]. However, we cannot exclude a priori that there are other causal agents, such as the Muenchen and Lora serovars. In fact, these serovars, together with Bratislava, have been isolated from the genital tracts and kidneys of sows that have abortions, from aborted fetuses and from boars in the USA [40], the Netherlands [41] and Northern Ireland [42,43]. Serogroup Australis has emerged as the major swine-maintained cause of *Leptospira* infections, but its epidemiological role remains poorly understood owing to isolation difficulties [44]. The isolation of serovar Bratislava still represents a challenge in the diagnosis of leptospirosis and, in fact, in contrast to the high seroprevalence reported worldwide [3,45,46,47], *L. interrogans* Bratislava has only been recovered from swine in a few countries, such as the United Kingdom [48], Germany [44], the USA [40,49,50], Vietnam [51] and Brazil [36]. Even in this study, we did not isolate any Bratislava strains. This may result from the quality and composition of the medium used, as well as the low bacterial load in kidneys, the organ used for the culture isolation [52]. In fact, Ellis et al. [52] found a higher isolation frequency for the Australis serogroup (Bratislava and Muenchen) from genital organs compared with kidneys. Therefore, improvements in the quality and composition of the isolation medium, as well as sampling procedures, must be considered. 

Pomona, the second most predominant serogroup in this work, is among the most common serogroup isolated from swine worldwide [53,54,55], and many strains of this serogroup are adapted to swine, which is recognized as a maintenance host [3]. Pigs infected with serogroup Pomona often experience abortions, stillbirths or the births of weak or ill piglets, and the infections result in subsequent decreased reproductive performances. Adult non-pregnant animals are usually asymptomatic carriers. Unfortunately, no data regarding the clinical symptoms of the analyzed pigs in this study were available. Swine infections caused by Pomona strains have been observed in Europe, particularly in its eastern and southern regions [56,57]. Recently, Bertelloni and colleagues [27] highlighted a recurrence of serogroup Pomona in pigs in North–Central Italy, revealing a Pomona serogroup seropositivity of 18% in the screened farms and of 8% in analyzed sera. Apart from swine, Pomona also causes clinically and economically significant infections in cattle, sheep and horses in many countries, resulting particularly in reproductive problems and equine uveitis [58]. A study from New Zealand reported that 31% of people working or residing on or within a 50-km radius of pig farms had microscopic agglutination titers of 1:24 or greater to one or more serovars of *L. interrogans*, with the majority being to Pomona [59]. Moreover, the ST140 genotype, the same as in our infected pigs, has been associated with symptomatic human leptospirosis in New Caledonia, Australia and Sri Lanka (this publication made use of the *Leptospira* MLST website https://pubmlst.org/Leptospira/ sited at the University of Oxford [25]. The development of this site has been funded by the Wellcome Trust).

For Tarassovi, historical data supported the widespread presence of this serogroup among the Italian pig population. In 1990, Tagliabue reported the isolation of four Tarassovi strains from swine kidneys [60], while in 1993, Scanziani and colleagues [37] described mild renal lesions in pigs affected by Tarassovi. Additionally, in 1995, Tagliabue and Farina reported a seroprevalence of 6.8% among the pig population, using a cut-off titer of 1:400 [38]. The identification of Tarassovi as the infective serogroup of swine in the past was corroborated by its presence in the vaccine formulation, indicating the need to protect hosts from this infective agent. In accordance with the observations already made by Tagliabue and Farina in 1995 [38], which noted a progressive decline in the Tarassovi frequency during the last few years, our study found that, in Italy, the infections caused by Tarassovi are decreasing, being responsible for the positivity of only 0.16% of the total samples tested. The infections caused by Tarassovi in swine have become incidental and probably result from contact with wild animals, including turtles, which act as a maintenance host for this serovar [61].

Among the minor serogroups found in tested pigs, we payed particular attention to some of them, discussed below. First of all, serogroup Icterohaemorrhagiae whose low prevalence could indicate the use of good rodent control measures inside and around the tested pig farms [62]. Moreover, the low prevalence observed for Grippotyphosa, Sejroe and Canicola were in agreement with historical data that described these serogroups as causes of incidental infection transmitted to pigs from wildlife hosts (serogroup Grippothyphosa [4]), from dogs (serogroup Sejroe serovar Sejroe [63] and serogroup Canicola serovar Canicola [64]), from bovines (serogroup Sejroe serovar Hardjo [4]) or small rodents (serogroup Sejroe serovar Saxkoebing [65]). 

Through genotyping techniques we found that all the isolated and extracted samples belonged to the Pomona serogroup but, interestingly, from 2014, new Pomona genotypes started to circulate in Northern Italy. Unfortunately, the Sequence Type provided by MLST analysis (ST140) being associated with isolates characterized as belonging to serogroups Pomona serovar Pomona, serogroup Pyrogenes serovar Guaratuba and serogroup Grippothyphosa (data from BIGSdb) did not permit any discrimination at the serogroup and at the serovar level, this ST In this case, the information provided by MLST genotyping is limited to species discrimination (i.e., *L. interrogans*). However, we should distinguish two different situations: in the case of isolated strains, since serological analysis by polyclonal anti-sera and by mAbs were possible, we were able to distinguish their serovar (Pomona) and serogroup (Pomona). In the case of DNA samples, we cannot assume that they are definitively serovar Pomona based only on the MLST results. However, we can assume that six of the eight samples belonged to serogroup Pomona serovar Pomona because their MLVA profiles were identical to that of strain Mezzano I. 

Interestingly, two isolates showed new mAb responses and new STs. Their reactions to the mAbs specific for the Pomona serogroup were very similar to each other but different from any of the known serogroup Pomona strain profiles. Their pattern was compatible with serovar Tsaratsovo, which was first isolated from harvest mice (*Micromys minutus*) in 1962, in the Plovdiv District of Bulgaria by Ivanov and later found in rodents in some parts of Europe [66]. Nevertheless, only mAbs F48 C6 and F61 C7, included in our panel, were tested on this serovar. Therefore, these data are insufficient to determine their serovars states. However, from an epidemiological point of view, this serovar has never been found in Italy; thus, it is very improbable that Tsaratsovo exists among the Italian pig population. Genotyping analyses of these strains indicated that they had the same VNTR pattern of Mozdok st. 5621 and belonged to ST288 and ST289, which had not been described previously, and are similar to ST117, which was found in *L. kirschneri* Mozdok st. 5621. In addition, the cgMLST analysis revealed that the most similar strains are classified as *Leptospira,* species *kirschneri,* serovar Mozdok. Definitely, MLST and cgMLST analyses excluded their belonging to the Tsaratsovo serovar, because strain Tsaratsovo B 81/7 was characterized as ST115 and cgST 245. We can also exclude Altodouro and Kunming, the other serovars included in *L. kirschneri* species and the Pomona serogroup, because they showed ST100 and ST70, respectively, with none of the seven loci being in common with those of ST288 and ST289. Therefore, we can assert that they are very similar to *L. kirschneri* Mozdok, but accurate descriptions of their genetic contents in comparison with serovar Mozdok are still being compiled. From an epidemiologic point of view, several studies have identified serovar Mozdok across Europe [67,68,69,70,71] in dogs [72] and in wild rodents [70,73,74]. It has been described also as a possible cause of abortions in cattle [75] and in pigs [69,73,74] and has been responsible of human leptospirosis in Cuba [76]. 

Owing to the advent of next-generation sequencing and the publication of the cgMLST scheme, the resolution powers of sequence-based analyses on *Leptospira* are increasing but the definitions of allelic distance cut-offs for the differentiation of strains are still open and in urgent need of cgMLST classification. 

Our study, which provides information on the *Leptospira* serovars present in the Italian pig population, is also useful for implementing a successful diagnostic and vaccination program. Commercial *Leptospira* vaccines are available globally for cattle, pigs and dogs, but vaccination has proven to be only partially effective, owing in part to the serovar-restricted nature of vaccine-induced immunity and the potential presence of local serovars other than those included in the vaccines. In Italy, prior to 2010, a trivalent vaccine against Bratislava (serogroup Australis), Pomona (Pomona) and Hyos (Tarassovi) was available for swine. It was used in breeding boars, in non-pregnant sows and in pregnant sows before the octave week of gestation. An unpublished survey carried out by the National Centre of Leptospirosis of IZSLER revealed that among 333 pig farms in Northern Italy during the period February 2004–December 2005, only 19% of the sampled farms adopted the vaccination practice. The reasons for this limited use of vaccination against leptospirosis in pigs included the risk that this disease is poorly understood by farmers and that leptospirosis is a secondary problem in the farm life/economy compared with other more virulent and economically important diseases. Nevertheless, since MAT does not discriminate between vaccination titers and titers due to exposure and because the vaccination status of pigs tested in this study prior to 2010 were unknown, the risk of having an altered number of positive pigs due to the interference of vaccination should be considered. About this aspect, the previously mentioned survey reported that in the farms where vaccination against *Leptospira* was practiced, 41% of pigs resulted as seropositive, while where it was not practiced seropositive animals were equal to only 9.8%. However, when considering this situation, it highlighted various scenarios: in the case of Australis, the interference of vaccination immunity was significant because the percentage of seropositives increased from 6.1% (of unvaccinated farms) to 33.9% (of vaccinated farms), while for Pomona and Tarassovi, the interference level was lower, going from 2.4% to 5.4% and from 1.2% to 5.4% for unvaccinated and vaccinated farms, respectively (data not published). Based on these data, we can exclude the interference of vaccination on our seropositivity results, given that the overall seropositivity and the seropositivity levels related to Australis, Pomona and Tarassovi (13.1%, 8.1%, 1.9% and 0.2%, respectively) were in agreement with the data from the unvaccinated farms reported in the survey (9%, 6.1%, 2.4% and 1.2%). Furthermore, in this study the trends in the seropositivity of Australis, Pomona and Tarassovi did not reveal any remarkable changes after the end of vaccine use in Italy occurred in 2010, which provided further evidence for our statement. 

Considering the serological data obtained and the frequency, in particular, of the Australis and Pomona serogroups, the use of vaccination in pig herds is highly advisable. In this regard, in March 2019, a vaccine has been registered in Italy. This vaccine contains nine serovars (Bananal, Bratislava, Canicola, Copenhageni, Gryppotyphosa, Icterohaemorrhagiae Pomona, Tarassovi, Vughia) and is active against six serogroups (Australis, Canicola, Gryppotyphosa, Icterohaemorrhagiae, Pomona, Tarassovi). As reported by Jacobs et al. [77], this vaccine can be safely used in gilts and sows and induces significant protection for the duration of at least one year. Furthermore, after a challenge with serovar Pomona, it induces protection against clinical signs, leptospiraemia and foetal death [78]. Its use can be considered a valid aid in limiting the infection among pig herds in our territory.

## 4. Materials and Methods 

### 4.1. Sampling

A total of 133,660 sera, 347 kidneys and 470 biological samples (from kidneys and unspecified tissues, as well as urine) were included in this study. These samples were collected over 16 years (from 2002 to 2017) from 4715 pig farms located in Northern Italy (Lombardy and Emilia Romagna regions) during the routine diagnostic activity. In these farms, testing for *Leptospira* infections was previously included in the disease-monitoring programs. The samples were collected and analyzed by the National Reference Centre for Animal Leptospirosis at the Istituto Zooprofilattico Sperimentale della Lombardia ed Emilia Romagna (IZSLER) located in Brescia (Italy). In this study, data already published by Tagliabue [61] collected in 2010 and 2011, were also included. In Table 8 we report the numbers of collected serum samples and farms. 

Starting from 2008, 347 well-preserved samples were submitted for culture-based causal agent isolation. Since 2009, when molecular methods for the routine diagnosis of leptospirosis were introduced, 470 clinical samples were analyzed using a LipL32-based real-time PCR assay.

To cover the entire analysis period, 43 randomly isolated samples and DNA extracted from 8 biological samples were submitted to genotyping analysis (Supplementary Table S2). For the isolation and genotyping procedures, we selected only one positive sample per farm, assuming that all the positive animals of the same herd were infected with the same strain.

### 4.2. Serological Tests of Serum Samples

Sera were examined for the presence of antibodies against pathogenic *Leptospira* using the MAT in accordance with OIE standards [11]. For MAT testing, live cultures of eight reference strains of *Leptospira* were used (Table 9). They were cultivated at 30 ± 1 °C in EMJH (Ellinghausen, McCullough, Johnson, Harris) medium enriched with bovine serum albumin supplement (10% v/v) [79,80]. Sera were pretested at the final dilution of 1/100. Sera with 50% agglutination were retested to determine an endpoint using dilutions of sera beginning at 1/100 through to 1/6400. Serum samples with the widely accepted minimum significant titer of 100 (reciprocal of the final dilution of serum with 50% agglutination) were assessed positive.

### 4.3. DNA Extraction and Real-Time PCR Detection

DNA was extracted from 0.5–4.0 mL of urine or tissue homogenate using the PureLink Genomic DNA kit (Invitrogen, Paisley, UK) according to the manufacturer’s instructions. An internal control DNA (0.1 µL per µL of elution volume) was added to the digestion buffer. A Taqman-based PCR assay targeting the lipL32 gene was used to detect pathogenic leptospires using primers described previously [17]. The PCR was performed in a 25-µL final volume, using 5 µL of extracted DNA, 5 µL of 5× Mastermix Quantifast (Quantifast Pathogen + IC Kit, Qiagen, Hilden, Germany), 700 nM of primers and 200 nM of the probe. All extraction session included a negative control (water) and all amplification sessions included both a negative (water) and a positive control (DNA of *Leptospira interrogans* Pomona). The assay was performed on a Bio-Rad CFX96 System using the following thermal conditions: a holding stage of 95 °C for 5 min, and 45 cycles of 95 °C for 15 s and 60 °C for 30 s. Supplementary Table S4 reported the interpretation criteria of Real-time PCR results. 

### 4.4. Isolation of Leptospira from Swine Kidneys

For isolation, 1 g of tissue was diluted, in a plastic bag, in 9 mL of EMJH added with 5-fluoruracil (selective EMJH) [79,80] and homogenized. This suspension was then filtered and collected in a sterile tube and diluted in a four ten-fold serial dilutions (10^−2^ to 10^−5^) performed in semisolid selective EMJH (added with 5-fluoruracil and agar). The cultures were incubated at 30 ± 1 °C and observed under dark-field microscopy weekly for up to 6 months. In case of contamination, the cultures were filtrated through a 0.22 μm sterile syringe filter and sub-cultured in fresh semisolid selective EMJH medium.

### 4.5. Characterization of Pig Isolates Using MAT

The serogroups of the isolates were determined by MAT using a panel of eight polyclonal anti-sera against the eight serovars described in Table 9. A high rate of agglutination with a particular antiserum was used to identify the presumptive serogroup of the strain [81].

Serovar classification was achieved by MAT using panels of mAbs directed against polysaccharide antigens, specific to the serogroup identified. A battery of five mAbs (F43 C9, F46 C9, F48 C6, F58 C1 and F61 C7), which react with serovars of the serogroup Pomona [82,83], were used to determine the serovar states of our isolates. These mAbs and the polyclonal anti-sera were previously purchased from the OIE Leptospirosis Reference Centre, Royal Tropical Institute (KIT), (Amsterdam, The Netherlands), and the protocol was in accordance to the standard serological methods used in this reference laboratory. The reference strains *L. interrogans* Pomona st. Pomona and *L. kirschneri* Mozdok st. 5621, provided by KIT, were used as controls for serovar identification. To interpret the results, we referred to the expected maximum dilution titer provided by the Leptospirosis Reference Centre of KIT, as reported in Table 10.

### 4.6. Genotyping

#### 4.6.1. MLST 

To genotype leptospires we used the scheme proposed by Bonsilp in 2013 [22], based on the seven housekeeping genes UDP-N-acetylglucosamine pyrophosphorylase (glmU), UDP-N-acetylglucosamine pyrophosphorylase (pntA), 2-oxoglutarate dehydrogenase E1 component (sucA), triosephosphate isomerase (tpiA), 1-phosphofructokinase (pfkB), rod shape-determining protein rodA (mreA) and acyl-CoA transferase/carnitine dehydratase (caiB). For the isolates, these loci were amplified using a KAPA2G Robust HotStart PCR kit (Kapa Biosystems Resnova, Roma, Italy) in a 25-µL total volume containing 0.4 µM each primer, 2.5 µL of boiled culture and MgCl2 at the following concentrations: 1.5 mM for mreA, pfkB, pntA, caiB and glmU; 2.5 mM for sucA; and 3.5 mM for tpiA. Temperature cycling was performed as follows: 1 cycle at 95 °C for 15 min, 35 amplification cycles of 95 °C, 55 °C for 30 s and 72 °C for 1 min, followed by a final elongation at 72 °C for 10 min. To genotype the DNA extracted from biological samples, we used the protocol described by Weiss and colleagues [23], consisting of one PCR reaction, similar to that described for isolates, but performed using 5 µL of extracted DNA, followed by a second amplification reaction, that used nested primers to improve the sensitivity. The nested PCR was performed in 25-μL reactions containing 5 pmol of each primer and 2 μL of the first-round PCR product. Cycling conditions were as follows: 95 °C for 10 min, 5 cycles of 95 °C for 30 s, 46 °C for 30 s and 72 °C for 30 s. In addition, 10 cycles, in which the annealing temperature increased by 1°C per cycle, and 20 cycles with an annealing temperature of 56 °C, were performed. A final extension step at 72 °C for 7 min was performed. The presence of PCR products was verified on 2% agarose gels stained with EuroSafe Nucleic Acid Stain (Euroclone, Milan, Italy). PCR products were purified using the NucleoSpin^®^ Gel and PCR Clean-up (Macherey-Nagel, Düren, Germany) or with exonuclease I and FastAp Thermosensitive Phospahatase alkaline (Thermo Fisher Scientific, Waltham, MA, USA) according to the manufacturer’s instructions. Cycle sequencing reactions were performed using the BigDye^®^ Terminator Cycle Sequencing kit version 1.1 (Applied Biosystems, Foster City, CA, USA). Reactions were purified using a BigDye^®^ XTerminatorTM Purification kit Thermo Fisher Scientific, Waltham, MA, USA) and sequenced on a Genetic Analyzer 3500xl sequencer (Thermo Fisher Scientific) according to the manufacturer’s instructions. Nucleotide sequences were assembled using the SeqMan module of the Lasergene sequencing analysis software package (DNASTAR, Inc., Madison, WI, USA). Assembled sequences were trimmed and aligned to allele reference sequences downloaded from the Bacterial Isolate Genome Sequence Database (BIGSdb) [25] (https://pubmlst.org/Leptospira/), to assign allele numbers to all seven loci. For strain identification, allelic profiles were queried against the *Leptospira* BIGSdb.

The phylogenetic tree was built using the concatemer of the seven MLST genes linked in the followed order: glmU-pntA-sucA-tpiA-pfkB-mreA-caiB using MEGA7 [84]. The phylogeny was inferred using the Neighbor-Joining method and by calculating genetic distances using the p-distance method.

#### 4.6.2. VNTR Analysis

Five discriminatory loci (VNTR-4, VNTR-7, VNTR-10, VNTR-Lb4 and VNTR-Lb5) were used to characterize the isolates and the extracted DNA samples as described by Salaün and colleagues in 2006 [21]. Briefly, 5 µL of boiled isolate or extracted DNA was added to 20 µL of the KAPA2G Robust HotStart PCR kit (Kapa Biosystems Resnova) reaction mixture that contained 10 pmol of each primer. The PCR was carried out as follows: 95 °C for 15 min and 35 cycles of 95 °C for 30 s, 55 °C for 30 s and 72 °C for 1 min. An additional extension for 10 min at 72 °C was added to the end of the run. The PCR products were analyzed on 2% agarose gels stained with EuroSafe Nucleic Acid Stain (Euroclone), and the molecular weights were estimated by comparison with a 100-bp DNA ladder (Thermo Fisher Scientific). If the length of PCR product was unclear, we sequenced it using the same sequencing protocol described for the MLST analysis.

#### 4.6.3. Genomic DNA Extraction from Cultured Strains

Genomic DNA of two *Leptospira* isolates from pigs belonging to new Sequence Types (ID 393 and 411) were extracted from the liquid EMJH cultures using the DNeasy Blood and Tissue kit (Qiagen), according to the manufacturer’s instructions. Extracted DNA was successfully used for species confirmation and for WGS. One strain isolated from pig previously identify as *L. interrogans* servar Pomona, “strain IZSLER 349/2007”, and the reference strain Mozdok 5621 were included as controls for the cgMLST analysis.

#### 4.6.4. Species Confirmation

The species assignments of the isolates 393 and 411 were confirmed by sequencing portions of their 16S rRNA genes using the Microseq 500 16S rDNA PCR kit (Applied Biosystems, Foster City, CA, USA). The obtained sequences were compared with a controlled and validated reference database (MicroseqID; Applied Biosystems) and through BLAST-based analyses to determine the species of the *Leptospira* strains.

#### 4.6.5. WGS and the cgMLST Analysis

WGS was performed on the four genomic DNAs extracted from cultured strains (ID 393, 411, IZSLER 349/2007 and strain Mozdok 5621) using an Illumina NextSeq (Illumina, San Diego, CA, USA) platform with a paired-end 150× 2-bp run starting from a genomic library prepared with a Nextera DNA Flex kit (Illumina). Raw reads were filtered using Trimmomatic ver. 0.38 [85], assembled using SPAdes Assembler ver. 3.9.0 [86] and evaluated using QUAST ver. 4.2 [87]. The resulting contigs were taxonomically classified using Kaiju software [88] and only the *Leptospira* contigs were used for further analyses. The draft genomes of samples 393, 411, IZSLER 349/2007 and strain Mozdok 5621 were used to perform cgMLST analyses with BIGSdb [25], using the cgMLST scheme recently developed by Guglielmini et al. [24].

Ethical statement: The study was exempt of ethical approval procedures because animal samplings were performed during the routinely diagnostic procedures in naturally infected farms.

## 5. Conclusions

In this study, an intensive serological survey evidenced that Australis and Pomona were the most serogroups causing leptospirosis in pigs in Italy. Molecular analyses revealed that a stable genotype of strains belonging to *L. interrogans* serogroup Pomona had been circulating for 10 years (from 2002 until 2013) and interestingly, *L. interrogans* strains collected from 2014 onwards had various heterogeneous genetic profiles. Furthermore, new strains belonging to *L. kirschneri* species have been identified for the first time. Since an accurate identification of the infective strain is crucial for an adequate vaccine formulation, these findings provide an important contribution for addressing prevention and intervention strategies to reduce infection risks on farms.

## Figures and Tables

**Table 1:** Numbers and percentages of swine serum samples that tested positive by Microscopic Agglutination Test (MAT) for Leptospira serogroups from 2002 to 2017. Only positivity to one serogroup was included.

Year	Serogroups								No. of Positive Samples
	A	B	C	G	I	P	S	T	
2002	1078	6	5	1	25	379	4	117	1615
2003	925	2	1	6	22	240	5	34	1235
2004	690	2			28	275	4	16	1015
2005	662	1	1		7	283	5	6	965
2006	301	7	2		9	440	7	5	771
2007	933	3	1	2	27	245	16	7	1234
2008	824	1			1	115	7	9	957
2009	647	5	1		6	157	1	2	819
2010	793		3		5	90	12		903
2011	639	2			6	52	1	1	701
2012	1414	3	8	2	7	64	10	1	1509
2013	334	2			11	35	6	1	389
2014	346	1	1		5	61	2		416
2015	441	5	2	2	6	29	9	3	497
2016	385	3	3		18	70	2	4	485
2017	261	1	3	3	10	16	1	3	298
No. of positive samples	10,673	44	31	16	193	2551	92	209	13,809
Percentage (%) of the positives (N = 13,809)	77.29	0.32	0.22	0.12	1.40	18.47	0.67	1.51	100
Percentage (%) of the total (N = 131,660)	8.11	0.03	0.02	0.01	0.15	1.94	0.07	0.16	10.49

A, Australis; B, Ballum; C, Canicola; G, Grippotyphosa; I, Icterohaemorrhagiae; P, Pomona; S, Sejroe; T, Tarassovi.

**Table 2:** Sera that react to more than one serogroup by Microscopic Agglutination Test (MAT).

Serogroups Combination	N° of Sera	Serogroups Combination	N° of Sera
A-P	1847	B-P	3
A-I-P	571	C-G-S	3
A-T	216	A-B-S	2
A-I	214	A-C-G	2
I-P	93	A-C-I-P-S	2
A-P-T	66	A-C-S	2
P-T	64	A-G-P-S	2
A-G-P	46	G-S	2
A-G-I-P	44	A-B-C-P	1
A-C-I-P	21	A-B-I-P-T	1
G-P	18	A-C-G-I-P-S-T	1
A-I-P-T	14	A-C-G-S	1
A-S	14	A-C-I-T	1
A-B-I-P	8	A-C-P	1
A-I-P-S	8	A-G-I-S	1
A-I-T	8	A-G-P-T	1
C-I	8	A-I-S	1
A-P-S	7	A-P-S-T	1
C-S	7	A-S-T	1
G-I-P	7	B-G	1
A-B	6	B-I	1
A-B-C-I-P	6	C-G	1
A-G	6	C-G-I	1
P-S	6	C-G-I-S	1
A-B-P	5	C-I-P	1
A-C-I	5	C-P	1
A-G-I	5	C-T	1
A-B-C-G-I-P-S-T	4	G-P-S	1
G-I	4	I-P-T	1
A-B-C	3	I-S	1
A-G-I-P-S	3	I-T	1
N. of total sera: 3375			

A, Australis; B, Ballum; C, Canicola; G, Grippotyphosa; I, Icterohaemorrhagiae; P, Pomona; S, Sejroe; T, Tarassovi.

**Table 3:** Detailed MAT titers of samples reacting to one serogroup.

Serogroup	Titer							Total
	1:100	1:200	1:400	1:800	1:1600	1:3200	1:6400	
Australis	6901	2836	694	170	43	16	5	10,665
Ballum	14	15	8	3	1	0	0	41
Canicola	22	7	1	0	1	0	0	31
Grippotyphosa	7	1	1	2	0	2	1	14
Icterohaemorragiae	115	29	16	6	0	0	0	166
Pomona	529	516	435	333	255	172	178	2418
Sejroe	35	23	19	6	6	0	1	90
Tarassovi	116	54	24	10	1	1	0	206

**Table 4:** Results of the MAT analysis of tested farms. Number (n) and percentage (%) of positive farms between 2002 and 2017.

Year	Number of Tested Farms	*Leptospira* Positive Farms	
		n	%
2002	452	226	50.00%
2003	455	243	53.41%
2004	528	244	46.21%
2005	478	252	52.72%
2006	613	140	22.84%
2007	332	190	57.23%
2008	265	162	61.13%
2009	246	146	59.35%
2010	225	155	68.89%
2011	202	150	74.26%
2012	298	231	77.52%
2013	135	78	57.78%
2014	130	79	60.77%
2015	128	88	68.75%
2016	128	82	64.06%
2017	101	62	61.39%
Total	4715	2528	53.62%

**Table 5:** Agglutination titers of Monoclonal Antibodies (mAbs) against the Pomona serogroup reference strains and the isolated samples from pigs (titers in reciprocal).

Species	Serogroup	Serovar	Strain	ID IZSLER *	mAbs				
					F43 C9	F46 C9	F48 C6	F58 C1	F61 C7
*L. interrogans*	Pomona	Pomona	Pomona	222	1280	0	5120	0	0
*L. kirschneri*	Pomona	Mozdok	5621	311	2560	10,240	2560	20,480	10,240
*L. interrogans*	Pomona	Pomona		321	320	0	640	0	0
*L. interrogans*	Pomona	Pomona		331	640	0	1280	0	0
*L. interrogans*	Pomona	Pomona		335	640	0	10,240	0	0
*L. interrogans*	Pomona	Pomona		340	320	0	2560	0	0
*L. interrogans*	Pomona	Pomona		349	160	0	1280	0	0
*L. interrogans*	Pomona	Pomona		354	640	0	5120	0	0
*L. interrogans*	Pomona	Pomona		362	2560	0	5120	0	0
*L. interrogans*	Pomona	Pomona		385	1280	0	5120	0	0
*L. interrogans*	Pomona	Pomona		391	2560	0	2560	0	0
*L. interrogans*	Pomona	Pomona		430	320	0	1280	0	0
unknown	Pomona	unclassified		393	1280	20,480	0	40,960	20,480
unknown	Pomona	unclassified		411	5120	40,960	0	40,960	20,480

*: The asterisk indicates the identification numbers of the isolates present in Istituto Zooprofilattico Sperimentale della Lombardia e dell’Emilia Romagna (IZSLER) collection.

**Table 6:** Core genome (cg) MLST analysis using the Bacterial Isolate Genome Sequence Database.

	Sample Strains			
	393	411	Mozdok 5621	IZSLER 349/2007
**Closest cgST(s) present in BIGSdb**	cgST200 cgST201	cgST106 cgST111 cgST200 cgST201	cgST106	cgST373 cgST376
**Loci matched with the closest cgST(s)**	524/545 (96.1%)	523/545 (96%)	523/545 (96%)	539/545 (98.9%)
Clonal group *	73	73	73	5

* Based on a cut-off value of 40 allelic differences to define clonal groups, as reported by Guglielmini et al. [24]

**Table 7:** Metadata of isolates having the most similar allelic profiles to those of our samples retrieved from BIGSdb.

cgST	ST *	ID	Isolate	Species	Serogroup	Country	Serovar	Host
106	117	134	Vehlefans 2	*L. kirschneri*	Pomona	Unknown	Mozdok	Cow
111	117	140	Vehlefans 3	*L. kirschneri*	Pomona	Portugal	Mozdok	Mouse
200	117	251	61H	*L. kirschneri*	Pomona	Brazil	Mozdok	Human
201	117	252	M36/05	*L. kirschneri*	Pomona	Brazil	Mozdok	Rat
373/376	140	455	201700301	*L. interrogans*	Pomona	Italy	unknown	Cow
373/376	140	456	201700306	*L. interrogans*	Pomona	Italy	unknown	Other mammal

* Using MLST scheme 1 developed by Boonsilp [22].

**Table 8:** Numbers of serum samples analyzed by MAT and farms involved in this study.

Year	Number of Serum Samples	Number of Farms
2002	18,096	452
2003	15,161	454
2004	10,881	528
2005	11,524	478
2006	11,296	613
2007	9474	332
2008	8059	265
2009	7693	246
2010	5841	225
2011	4825	202
2012	9330	298
2013	4029	135
2014	4306	130
2015	4055	128
2016	4585	128
2017	2505	101
Total	131,660	4715

**Table 9:** Panel of eight *Leptospira spp*. used as live antigens for MAT.

Species	Serogroup	Serovar	Strain
*L. interrogans*	Australis	Bratislava	Riccio 2
*L. borgpetersenii*	Ballum	Ballum	Mus 127
*L. interrogans*	Canicola	Canicola	Alarik
*L. kirschneri*	Grippotyphosa	Grippotyphosa	Moskva V
*L. interrogans*	Icterohaemorrhagiae	Copenhageni	Wijnberg
*L. interrogans*	Pomona	Pomona	Pomona
*L. interrogans*	Sejroe	Hardjo	Hadjoprajitno
*L. borgpetersenii*	Tarassovi	Tarassovi	Mitis-Johnson

**Table 10:** Maximum dilution titer patterns of the serovars in the Pomona serogroup against the available mAbs provided by the Leptospirosis Reference Centre, Royal Tropical Institute, Amsterdam, The Netherlands.

Species	Serogroup	Serovar	Strain	mAbs				
				F43 C9	F46 C9	F48 C6	F58 C1	F61 C7
*L. interrogans*	Pomona	Pomona	Pomona	1,280	0	5120	0	0
*L. kirschneri*	Pomona	Mozdok	5621	2,560	10,240	2560	20,480	10,240
*L. kirschneri*	Pomona	Altodouro	RIM 139	80	20,480	5120	20,480	20,480
*L. kirschneri*	Pomona	Tsaratsovo	B 81/7	nt	nt	0	nt	20,480
*L. kirschneri*	Pomona	Kunming	K5	640	2560	2560	640	2560
*L. borgpetersenii*	Pomona	Kunming	200901118	nt	nt	nt	nt	nt
*L. interrogans*	Pomona	Proechymis	1161 U	80	2560	640	40,960	10,240
*L. santarosai*	Pomona	Tropica	CZ 299	640	10,240	5120	1280	10,240

nt: not tested.

## Supplementary Materials

The following are available online at https://www.mdpi.com/2076-0817/9/5/332/s1, Table S1: Trends of outbreaks observed in 2002–2017 (only single positivities were considered), Table S2: Samples submitted for genotyping analysis, Table S3: Loci showing new alleles in samples 393 and 411. Table S4: Interpretation of Real-time PCR results. Common loci having the same new alleles in samples 393 and 411 are shown in bold, File S1: Comparison of allelic profiles resulting from the cgMLST analysis.
