# Supplementary material for: Serological Survey and Molecular Typing Reveal New Leptospira Serogroup Pomona Strains among Pigs of Northern Italy
Source: Pathogens. 2020 Apr 29;9(5):332. doi: 10.3390/pathogens9050332 (PMC7281294; doi:10.3390/pathogens9050332)
Supplement: Supplementary file 1 [file pathogens-09-00332-s001.zip › pathogens-777638/Supplementary Table revised manuscript/Table S1.docx]

**Table S1**. Trend of outbreaks observed in 2002-2017 (only single positivities were considered)

| **Serogroup** | **Year of sampling** | | | | | | | | | | | | | | | | **Mean** |
| --- | --- | --- | --- | --- | --- | --- | --- | --- | --- | --- | --- | --- | --- | --- | --- | --- | --- |
|  | **2002** | **2003** | **2004** | **2005** | **2006** | **2007** | **2008** | **2009** | **2010** | **2011** | **2012** | **2013** | **2014** | **2015** | **2016** | **2017** |  |
| **A** | 66,34% | 75,00% | 68,46% | 76,84% | 69,61% | 76,85% | 76,30% | 77,54% | 77,57% | 77,40% | 82,23% | 77,22% | 78,50% | 77,84% | 80,14% | 76,04% | 75,87% |
| **P** | 0,97% | 0,42% | 0,83% | 0,37% | 2,45% | 0,96% | 1,42% | 1,07% | 0,00% | 0,00% | 1,05% | 2,53% | 2,80% | 0,60% | 0,71% | 1,04% | 1,08% |
| **B** | 0,49% | 0,21% | 0,00% | 0,37% | 0,98% | 0,32% | 0,00% | 0,53% | 1,40% | 0,00% | 0,35% | 3,16% | 0,93% | 0,60% | 0,71% | 3,13% | 0,82% |
| **C** | 0,16% | 0,42% | 0,00% | 0,00% | 0,00% | 0,64% | 0,95% | 0,00% | 0,00% | 0,00% | 0,00% | 1,27% | 0,00% | 0,00% | 0,00% | 3,13% | 0,41% |
| **G** | 3,07% | 2,54% | 5,39% | 1,47% | 3,92% | 5,14% | 3,32% | 3,21% | 2,80% | 3,37% | 1,05% | 1,90% | 3,74% | 4,19% | 2,84% | 8,33% | 3,52% |
| **I** | 16,83% | 15,89% | 21,58% | 18,01% | 18,14% | 12,22% | 14,69% | 14,97% | 16,82% | 15,87% | 13,24% | 11,39% | 14,02% | 13,77% | 11,35% | 4,17% | 14,56% |
| **S** | 0,65% | 1,06% | 1,24% | 1,10% | 2,45% | 2,57% | 1,90% | 2,14% | 1,40% | 2,40% | 1,74% | 1,90% | 0,00% | 1,80% | 2,84% | 1,04% | 1,64% |
| **T** | 11,49% | 4,45% | 2,49% | 1,84% | 2,45% | 1,29% | 1,42% | 0,53% | 0,00% | 0,96% | 0,35% | 0,63% | 0,00% | 1,20% | 1,42% | 3,13% | 2,10% |
