# Supplementary material for: Serological Survey and Molecular Typing Reveal New Leptospira Serogroup Pomona Strains among Pigs of Northern Italy
Source: Pathogens. 2020 Apr 29;9(5):332. doi: 10.3390/pathogens9050332 (PMC7281294; doi:10.3390/pathogens9050332)
Supplement: Supplementary file 1 [file pathogens-09-00332-s001.zip › pathogens-777638/Supplementary Table revised manuscript/Table S2.docx]

**S2 Table.** Samples submitted to genotyping analysis

| **No.** | **ID** | **Internal Code** | **Type** | **Year of sampling** | **Region** | **Provence** |
| --- | --- | --- | --- | --- | --- | --- |
| 1 | 319 | 2002/24444/1 | isolate | 2002 | Emilia Romagna | MO |
| 2 | 320 | 2002/100296/1 | isolate | 2002 | Emilia Romagna | MO |
| 3 | 321 | 2002/198060/1 | isolate | 2002 | Lombardy | MN |
| 4 | 323 | 2003/25766/1 | isolate | 2003 | Emilia Romagna | MO |
| 5 | 327 | 2004/244848/3 | isolate | 2004 | Lombardy | MN |
| 6 | 330 | 2005/82950/1 | isolate | 2005 | Emilia Romagna | MO |
| 7 | 331 | 2005/261881/6 | isolate | 2005 | Lombardy | MN |
| 8 | 332 | 2005/281137/1 | isolate | 2005 | Lombardy | MN |
| 9 | 333 | 2006/10635/5 | isolate | 2006 | Lombardy | MN |
| 10 | 334 | 2006/29074/1 | isolate | 2006 | Emilia Romagna | MO |
| 11 | 335 | 2006/89288/2 | isolate | 2006 | Lombardy | MN |
| 12 | 336 | 2006/108702/5 | isolate | 2006 | Lombardy | MN |
| 13 | 337 | 2006/131109/1 | isolate | 2006 | Lombardy | MN |
| 14 | 338 | 2006/188883/4 | isolate | 2006 | Lombardy | MN |
| 15 | 339 | 2006/188887/2 | isolate | 2006 | Lombardy | MN |
| 16 | 340 | 2006/271763/2 | isolate | 2006 | Lombardy | MN |
| 17 | 341 | 2006/66864/1 | isolate | 2006 | Emilia Romagna | BO |
| 18 | 342 | 2011/158875/6 | isolate | 2011 | Lombardy | MN |
| 19 | 344 | 2011/202468/5 | isolate | 2011 | Lombardy | MN |
| 20 | 345 | 2007/11447/4 | isolate | 2007 | Lombardy | MN |
| 21 | 347 | 2007/207007/3 | isolate | 2007 | Emilia Romagna | RA |
| 22 | 349 | 2007/230836/5 | isolate | 2007 | Lombardy | MN |
| 23 | 350 | 2007/234297/1 | isolate | 2007 | Emilia Romagna | MO |
| 24 | 352 | 2007/335262/4 | isolate | 2007 | Emilia Romagna | RA |
| 25 | 354 | 2009/200947/2 | isolate | 2009 | Lombardy | MN |
| 26 | 362 | 2010/114428/2 | isolate | 2010 | Emilia Romagna | RA |
| 27 | 363 | 2010/162649/9 | isolate | 2010 | Emilia Romagna | RA |
| 28 | 383 | 2008/71661/5 | isolate | 2008 | Lombardy | MN |
| 29 | 386 | 2008/144920/1 | isolate | 2008 | Emilia Romagna | RA |
| 30 | 387 | 2008/159769/2 | isolate | 2008 | Lombardy | MN |
| 31 | 388 | 2008/169601/1 | isolate | 2008 | Emilia Romagna | RA |
| 32 | 391 | 2013/30290/1 | isolate | 2013 | Lombardy | MN |
| 33 | 392 | 2013/30361/1 | isolate | 2013 | Lombardy | MN |
| 34 | 393 | 2013/102621/1 | isolate | 2013 | Emilia Romagna | RA |
| 35 | 394 | 2013/106953/1 | isolate | 2013 | Lombardy | MN |
| 36 | 410 | 2014/5316/2 | isolate | 2014 | Emilia Romagna | MO |
| 37 | 411 | 2014/252492/2 | isolate | 2014 | Emilia Romagna | MO |
| 38 | 429 | 2015/98275/1 | isolate | 2015 | Lombardy | MN |
| 39 | 430 | 2015/160244/2 | isolate | 2015 | Lombardy | MN |
| 40 | 431 | 2015/293603/5 | isolate | 2015 | Lombardy | MN |
| 41 | 433 | 2016/61597/2 | isolate | 2016 | Lombardy | MN |
| 42 | 442 | 2017/38224/1 | isolate | 2017 | Lombardy | MN |
| 43 | 465 | 2017/297896/5 | isolate | 2017 | Lombardy | MN |
| 44 | 131225/3 |  | extracted DNA | 2016 | Lombardy | PV |
| 45 | 6120/9 |  | extracted DNA | 2017 | Lombardy | LO |
| 46 | 43994/1 |  | extracted DNA | 2017 | Lombardy | CR |
| 47 | 27177/1 |  | extracted DNA | 2017 | Emilia Romagna | RA |
| 48 | 12754/1 |  | extracted DNA | 2017 | Emilia Romagna | RA |
| 49 | 11509/15 |  | extracted DNA | 2016 | Emilia Romagna | RA |
| 50 | 115560/1 |  | extracted DNA | 2017 | Emilia Romagna | MO |
| 51 | 224485/1 |  | extracted DNA | 2017 | Lombarby | LO |
