# Supplementary material for: Serological Survey and Molecular Typing Reveal New Leptospira Serogroup Pomona Strains among Pigs of Northern Italy
Source: Pathogens. 2020 Apr 29;9(5):332. doi: 10.3390/pathogens9050332 (PMC7281294; doi:10.3390/pathogens9050332)
Supplement: Supplementary file 1 [file pathogens-09-00332-s001.zip › pathogens-777638/Supplementary Table revised manuscript/Table S3.docx]

**Table S3.** Loci showing new alleles in sample 393 and 411. In bold are indicated the shared loci.

| **Sample 393** | |
| --- | --- |
| **Locus** | **Product** |
| **LIC_RS01605** | **glycine dehydrogenase (decarboxylating)** |
| **LIC_RS02715** | **transcriptional regulator** |
| LIC_RS03900 | DNA-directed RNA polymerase subunit beta' |
| **LIC_RS06005** | **SAM-dependent methyltransferase** |
| **LIC_RS06745** | **acetyl-CoA acetyltransferase** |
| **LIC_RS07025** | **endoflagellar motor switch protein** |
| **LIC_RS07470** | **membrane protein** |
| **LIC_RS07920** | **RNA polymerase sigma-54 factor** |
| **LIC_RS09025** | **3-isopropylmalate dehydrogenase** |
| **LIC_RS09245** | **ATP-dependent Clp protease ATP-binding subunit ClpA** |
| **LIC_RS12660** | **dihydrolipoamide dehydrogenase** |
| **LIC_RS13755** | **ABC transporter permease** |
| LIC_RS14075 | NADH dehydrogenase |
| LIC_RS17405 | ABC transporter permease |
| **LIC_RS17510** | **acetyl-CoA synthetase** |
| **LIC_RS17965** | **tRNA uridine 5-carboxymethylaminomethyl modification enzyme MnmG** |
| **LIC_RS18435** | **diphosphate-fructose-6-phosphate 1-phosphotransferase** |
| **Sample 411** | |
| **LIC_RS01605** | **glycine dehydrogenase (decarboxylating)** |
| **LIC_RS02715** | **transcriptional regulator** |
| LIC_RS04520 | Fe-S-cluster-containing hydrogenase |
| LIC_RS05180 | anti-sigma factor antagonist |
| **LIC_RS06005** | **SAM-dependent methyltransferase** |
| **LIC_RS06745** | **acetyl-CoA acetyltransferase** |
| **LIC_RS07025** | **endoflagellar motor switch protein** |
| **LIC_RS07470** | **membrane protein** |
| **LIC_RS07920** | **RNA polymerase sigma-54 factor** |
| LIC_RS08210 | hypothetical protein |
| **LIC_RS09025** | **3-isopropylmalate dehydrogenase** |
| **LIC_RS09245** | **ATP-dependent Clp protease ATP-binding subunit ClpA** |
| **LIC_RS12660** | **dihydrolipoamide dehydrogenase** |
| **LIC_RS13755** | **ABC transporter permease** |
| LIC_RS15980 | glucose-6-phosphate isomerase |
| LIC_RS16825 | two-component system sensor histidine kinase |
| **LIC_RS17510** | **acetyl-CoA synthetase** |
| **LIC_RS17965** | **tRNA uridine 5-carboxymethylaminomethyl modification enzyme MnmG** |
| **LIC_RS18435** | **diphosphate-fructose-6-phosphate 1-phosphotransferase** |
