# Supplementary material for: Serological Survey and Molecular Typing Reveal New Leptospira Serogroup Pomona Strains among Pigs of Northern Italy
Source: Pathogens. 2020 Apr 29;9(5):332. doi: 10.3390/pathogens9050332 (PMC7281294; doi:10.3390/pathogens9050332)
Supplement: Supplementary file 1 [file pathogens-09-00332-s001.zip › pathogens-777638/Supplementary Table revised manuscript/Table S4.docx]

**Table S4.** Interpretation of Real-time PCR results.

| **Expected results of controls** | | |
| --- | --- | --- |
| **Controls** | **CT _LipL32_** | **CT _IC_** |
| **Extraction negative control** | Absence or CT ≥ 40 | 26-36 |
| **PCR negative control** | Absence or CT ≥ 40 | Absence or Ct ≥ 40 |
| **PCR positive control** | 27≤ CT≤ 33 | 26 – 36 / ≥ 40 |
| **Interpretation of unknow sample result** | | |
| **Outcome** | **CT _LipL32_** | **CT _IC_** |
| **Presence of patogenic *Leptospira* spp.** | 5≤ CT≤ 38 | 26 – 36 / ≥ 40 |
| **Absence of pathogenic *Leptospira* spp.** | Absence or CT ≥ 40 | 26 – 36 |
| **Doubtful presence of pathogenic *Leptospira* spp.** | 38<CT≤40 | 26 – 36 |
| **Not conclusive result** | 38<CT≤40 | CT> 36 |
